# Molecular Mechanism of Sirtuin 1 Inhibition by Human Immunodeficiency Virus 1 Tat Protein

**DOI:** 10.3390/life13040949

**Published:** 2023-04-04

**Authors:** Ramona S. Adolph, Eileen Beck, Kristian Schweimer, Andrea Di Fonzo, Michael Weyand, Paul Rösch, Birgitta M. Wöhrl, Clemens Steegborn

**Affiliations:** 1 Department of Biochemistry, University of Bayreuth, 95440 Bayreuth, Germany; 2 Department of Biopolymers, University of Bayreuth, 95440 Bayreuth, Germany

**Keywords:** deacetylase, HIV, Sirt1, regulation, crystal structure

## Abstract

Sirtuins are NAD^+^-dependent protein lysine deacylases implicated in metabolic regulation and aging-related dysfunctions. The nuclear isoform Sirt1 deacetylates histones and transcription factors and contributes, e.g., to brain and immune cell functions. Upon infection by human immunodeficiency virus 1 (HIV1), Sirt1 deacetylates the viral transactivator of transcription (Tat) protein to promote the expression of the viral genome. Tat, in turn, inhibits Sirt1, leading to the T cell hyperactivation associated with HIV infection. Here, we describe the molecular mechanism of Tat-dependent sirtuin inhibition. Using Tat-derived peptides and recombinant Tat protein, we mapped the inhibitory activity to Tat residues 34–59, comprising Tat core and basic regions and including the Sirt1 deacetylation site Lys50. Tat binds to the sirtuin catalytic core and inhibits Sirt1, Sirt2, and Sirt3 with comparable potencies. Biochemical data and crystal structures of sirtuin complexes with Tat peptides reveal that Tat exploits its intrinsically extended basic region for binding to the sirtuin substrate binding cleft through substrate-like β-strand interactions, supported by charge complementarity. Tat Lys50 is positioned in the sirtuin substrate lysine pocket, although binding and inhibition do not require prior acetylation and rely on subtle differences to the binding of regular substrates. Our results provide mechanistic insights into sirtuin regulation by Tat, improving our understanding of physiological sirtuin regulation and the role of this interaction during HIV1 infection.

## 1. Introduction

Sirtuins are NAD^+^-dependent protein lysine deacylases that regulate a wide range of functions, from gene transcription and energy metabolism to stress responses and aging processes [1]. Among the seven human sirtuin isoforms (Sirt1–7), Sirt1 is best characterized. It is mainly located in the nucleus and acts as a deacetylase of histones and several transcription factors, such as p53 and PGC-1α, and it has been implicated in the regulation of cell proliferation, energy metabolism, and neuronal and immune cell function [1]. Sirt1 also contributes to the regulation of the amplification of human immunodeficiency virus 1 (HIV1), which infects CD4-carrying cells, in particular T helper cells, and thereby facilitates opportunistic infections and the development of acquired immunodeficiency syndrome (AIDS) [2,3]. Once the HIV capsid has entered the cell, the viral RNA genome is reverse-transcribed and integrated into the host genome. Transcription of viral genes is, then, activated by the HIV protein Tat (transactivator of transcription), and the 86–101 residue protein (depending on virus subtype) that binds to the transactivation response (TAR) element of the viral RNA transcript [4]. Tat comprises an N-terminal transactivation domain (residues 1–48), including a Cys-rich region (22–37) that can coordinate two Zn^2+^ ions, a core region (38–48), a basic, Arg-rich motif (ARM) implicated in nuclear localization and TAR binding (49–57), a Glu-rich segment (58–73), and a C-terminal region (74–86) [5]. Tat forms a complex with the p-TEFb (positive-acting transcription elongation factor) complex, TAR RNA, and RNA polymerase II for transcription activation, and it can further interact with a variety of proteins, such as the transcriptional regulator AP-4, the histone acetyltransferase p300, and Sirt1 [5,6,7]. Sirt1 was reported to deacetylate Tat and act as a coactivator [8]. While the inverse is the same, where Tat can inhibit Sirt1, a negative regulator of T cell activation, to induce hyperactivation [9], and to promote apoptosis of non-infected cells [10].

Sirt1 features the conserved sirtuin catalytic core of ~270 residues, which comprises a Rossman-fold domain and a smaller Zn^2+^-binding module [11,12]. A cleft between these subdomains accommodates NAD^+^ and recognizes the acylated substrate polypeptide with low sequence selectivity, and binding of these substrates is accompanied by Sirt1 closure movements [12,13,14,15]. The unique, NAD^+^-dependent sirtuin deacylation mechanism renders them metabolic sensors and proceeds via an alkylimidate between acyl lysine and nucleotide ribose, accompanied by the release of NAD^+^’s nicotinamide (NAM) moiety [16,17]. Rearrangement into a bicyclic intermediate and hydrolysis yield the products, deacylated polypeptide, and 2′-O-acyl-ADP-ribose. Isoform-specific N- and C-terminal extensions of the core contribute to cellular localization and regulation [18,19]. In Sirt1, the extensions are rather long and, mostly, dynamic, yet also comprise a so-called “essential for Sirt1 activity” (ESA) region and a sirtuin-activating compound (STAC) binding-domain (SBD) [20,21,22]. Several proteins have been reported to regulate Sirt1, namely the activator AROS (active regulator of Sirt1) and the inhibitors HIC1 (hypermethylated in cancer), Dbc1 (deleted in breast cancer), and HIV1 Tat. However, the mechanisms they employ are poorly understood. Initial characterization of the Sirt1/Tat interaction revealed a regulatory effect of Sirt1 on Tat [8]. Tat can be acetylated at Lys50, which is required for transactivation and leads to the binding of the p300/CBP-associated factor (PCAF) and dissociation of the p-TEFb component cyclinT1 [23,24]. Sirt1 deacetylates Tat-Lys50 and stimulates transactivation, possibly by recycling deacetylated Tat from elongation complexes for the assembly of new Tat/TAR/p-TEFb complexes [8]. Interestingly, Tat-Lys50 acetylation is not required for interaction with Sirt1, and a later report, in fact, described Tat as a potent inhibitor that targets the Sirt1 catalytic core (IC_50_ 3.5 µM) and was speculated to remain bound after being deacetylated to block the active site [9]. The molecular basis and exact interplay of the mutual regulatory effects of Tat and Sirt1 remain to be clarified, however.

Here, we report a biochemical and structural characterization of the inhibition of Sirt1 by HIV1 Tat. Tat inhibits Sirt1 potently in vitro; therefore, using full-length Tat and Sirt1 as well as fragments of both proteins allowed the mapping of the interaction to the Tat core and basic regions, and the Sirt1 catalytic core, respectively. Tat inhibition is competitive with the acyl peptide substrate and applicable to other sirtuin isoforms. Using the comparably inhibited Sirt3 as a model system, we solved a crystal structure of a sirtuin/Tat complex, revealing a substrate-like binding of the Tat core and basic region with an additional electrostatic component and subtle differences to regular substrates. Our results provide a mechanistic basis for Sirt1 inhibition by Tat and novel insights into the mutual regulation of the two proteins.

## 2. Results

### 2.1. Tat Inhibits Directly the Catalytic Core of Sirt1 and Also Those of Other Sirtuin Isoforms

**Figure 1:**
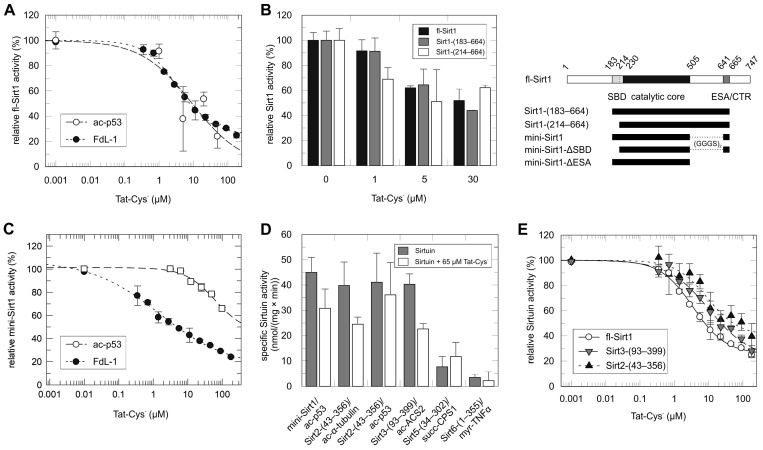
Sirt1 inhibition by Tat and mapping of the interaction sites. (**A**) Titration of fl-Sirt1 with Tat-Cys^−^ in a coupled enzymatic assay at 100 µM substrate (○) and an FdL assay at substrate K_m_ (●) (error bars: s.d., *n* = 2 in coupled assay, *n* = 3 in FdL assay). (**B**) Dose-dependent inhibition of various Sirt1 constructs by Tat-Cys^−^ at 100 µM FdL-1 (black: full-length Sirt1; gray: Sirt1-183–664; white: Sirt1-214–664). Sirt1 regions included in the respective construct are illustrated in the scheme on the right (error bars: s.d., *n* = 2). (**C**) Inhibition of mini-Sirt1 by Tat-Cys^−^ at substrate K_m_ in a coupled-enzyme assay (□) and an FdL assay (●), respectively, (error bars: s.d., *n* = 3–5). (**D**) Deacylation activity of human Sirtuin isoforms at substrate K_m_ in absence of Tat-Cys^−^ (gray) or in presence of 65 μM Tat-Cys^−^ (white). Sirt1,2,3 tested against acetylated peptides, Sirt5 against succinylated, and Sirt6 against myristoylated peptides (error bars: s.d., *n* = 3). (**E**) Tat-Cys^−^ titration of fl-Sirt1 (○), Sirt2 (▲), and Sirt3 (▼) in an FdL assay at substrate K_m_ (error bars: s.d., *n* = 3).

We first analyzed the effects of the 86-residue full-length Tat protein from the HIV1 Zaire 2 isolate (HIV1–Z2) on the activity of purified recombinant human full-length Sirt1 (fl-Sirt1). Sirt1-dependent deacetylation of a peptide derived from the physiological Sirt1 substrate p53 was monitored in a coupled enzymatic assay that measures the nicotinamide product of the sirtuin-catalyzed reaction [17]. A titration of fl-Sirt1 with recombinant full-length Tat-Cys^−^ (Tat variant with its 7 Cys residues replaced by Ser/Ala [25]) revealed an inhibition with an IC_50_ of ~20 μM (Figure 1A). Previously, a slightly higher potency had been reported (IC_50_ 3.5 μM [9]), which might be due to our use of the Tat-Cys^−^ variant or the previous use of the non-physiological, fluorophore-modified Fluor-de-Lys (FdL) deacetylation substrate. The short FdL substrate enables sensitive monitoring of its deacetylation via an increase in fluorescence, yet has a lower apparent affinity than regular peptides [17,26]. Repeating the full-length Tat-Cys^−^ titration with the lower-affinity FdL substrate and fluorescence monitoring indeed yielded an IC_50_ of 3.6 ± 0.8 μM (Figure 1A), matching excellently the value previously reported for Sirt1 inhibition by wildtype Tat (3.5 μM) in the same assay [9]. These data confirm that Tat binds directly to Sirt1 and inhibits the enzyme also against regular substrate peptides, and they show that Tat’s Cys residues—and thus its optional metal cofactors—are not relevant for the inhibitory effect and the potency. The dependency of the inhibition potency on the apparent affinity of the substrate used instead might indicate a peptide competitive component for inhibition.

Next, we tested Sirt1 deletion constructs in activity assays for their response to Tat, to analyze whether the catalytic domain is sufficient for inhibition. Deleting the flexible N-terminal domain (residues 1–182; Figure 1B), alone or in combination with the activator-binding SBD (residues 183–213), resulted in a similar inhibition of basal activity (Figure 1B). The so-called mini-Sirt1 construct comprising activator binding domain, catalytic core, artificial linker, and ESA region [27], indeed, showed quantitatively comparable inhibition effects: Tat-Cys^−^ titrations in assays with p53 substrate yielded an IC_50_ of ~30–40 µM and in FdL assays an IC_50_ of 2.3 ± 0.9 μM (Figure 1C). Cleaving off either the SBD or the ESA domain from mini-Sirt1 (mini-Sirt1-∆SBD and mini-Sirt1-∆ESA, respectively) resulted in unchanged inhibitory effects (Supplementary Figure S1A), showing that the SBD and ESA regions have no significant influence on Tat-dependent inhibition, which is consistent with interaction data from coimmunoprecipitation experiments [9].

Since only the catalytic domain is essential for Tat-dependent inhibition and Sirt1–7 share homolog catalytic domains, we analyzed whether Tat would also inhibit other human sirtuin isoforms. We find that Tat has pronounced inhibitory effects on Sirt2 and Sirt3 in both, continuous assays (Figure 1D) and FdL assays (IC_50_ 11.0 ± 4.4 µM for Sirt2 and 15.9 ± 10.7 µM for Sirt3; Figure 1E), consistent with previous deacetylation tests with immunoprecipitated sirtuins [8]. For Sirt5 and Sirt6, in contrast, no statistically significant effect was observed (Figure 1D), possibly due to structural differences in detail related to their different acyl preferences (see discussion). Thus, we conclude that the catalytic cores of the sirtuin isoforms with prominent deacetylase activity are amenable to potent inhibition by the HIV1 Tat protein.

### 2.2. Mapping of the Tat Region Inhibiting Sirt1

**Figure 2:**
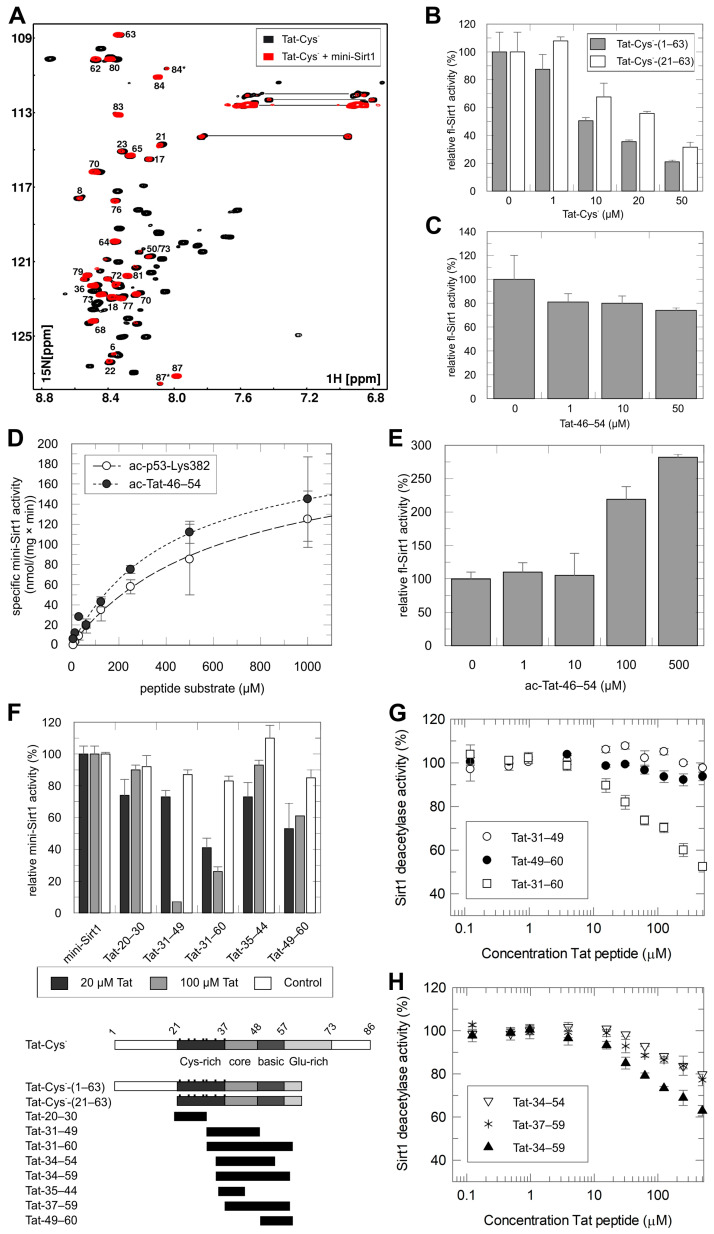
Mapping and characterization of the Tat region required for sirtuin inhibition. (**A**) Comparison of 2D-(^1^H, ^15^N)-HSQC spectra of 58 µM Tat-Cys^−^ before (●) and after (●) addition of mini-Sirt1 (2-fold molar excess). Remaining signals of backbone amides are labeled by sequence numbers. Signals with * denote a second amino acid conformation, signals connected via a horizontal line refer to the sidechain-NH_2_ of Gln/Asn. (**B**) Dose-dependent inhibition of fl-Sirt1 by the truncated Tat variants Tat-Cys^−^-(1–63) (gray) and Tat-Cys^−^-(21–63) (white) in an FdL assay at 100 µM FdL-1 (error bars: s.d., *n* = 2). (**C**) Dose-dependent inhibition of fl-Sirt1 by Tat-46–54 in a coupled-enzyme assay at 100 µM ac-p53 (error bars: s.d., *n* = 2). (**D**) Michaelis–Menten kinetics for deacetylation of ac-p53-Lys382 (○) and ac-Tat-46–54 (●) peptides by mini-Sirt1 (error bars: s.d., *n* = 3). (**E**) Dose-dependent increase of fl-Sirt1 activity by ac-Tat-46–54, whose addition increases the total substrate concentration, in a coupled-enzyme assay at 100 µM ac-p53 as the basal substrate (error bars: s.d., *n* = 2). (**F**) Dose-dependent inhibition of mini-Sirt1 by synthetic Tat peptides in an FdL assay at substrate K_m_. Control refers to Tat addition after stopping the reaction. Tat regions covered by peptides are indicated at the bottom (error bars: s.d., *n* = 3). (**G**) Titration of mini-Sirt1 with Tat-31–49, Tat-49–60, and Tat-31–60 in an FdL assay at substrate K_m_ (error bars: s.d., *n* = 3). (**H**) Titration of mini-Sirt1 with Tat-34–54, Tat-34–59, and Tat-37–59 in an FdL assay at substrate K_m_ (error bars: s.d., *n* = 3).

To identify the Tat regions involved in Sirt1 inhibition, we analyzed their interaction through 2D-NMR spectroscopy. We titrated unlabeled mini-Sirt1 to ^15^N-labeled Tat-Cys^−^ and analyzed 2D-(^1^H, ^15^N)-HSQC NMR spectra for changes in chemical shifts (Figure 2A), which indicate changes in an atom’s environment upon complex formation. Sirt1 addition caused a large number of HSQC signals to disappear, however, which is attributed to line broadening being beyond detection due to the formation of a larger complex or chemical exchange processes. The remaining, unchanged signals can be assigned mostly to Tat residues 63–87. This result suggests that the Tat C-terminal region is not relevant for binding to Sirt1. Thus, we tested N- and C-terminally shortened Tat-Cys^−^ constructs for Sirt1 inhibition. A construct comprising Tat-Cys^−^ residues 1–63, indeed, inhibited Sirt1 with an IC_50_ of ~10 µM, whereas a construct also lacking the N-terminal region from aa 1–20 repressed Sirt1 with an only slightly lower potency of ~30 µM (Figure 2B). Thus, we concluded that the region from residues 21–63 of Tat-Cys^−^ was sufficient for Sirt1 inhibition.

To further narrow down the Sirt1 inhibition part(s) of Tat, we used synthetic fragments of the Sirt1-inhibitory Tat region 21–63. We first tested Tat-46–54, which is centered on the physiological Tat acetylation site Lys50. Initial studies had indicated that Tat-Lys50 acetylation was not required for Sirt1 binding [8], although it was later proposed that Tat inhibited by remaining bound to the active site after Lys50 deacetylation [9]. Adding non-acetylated Tat-46–54 to a Sirt1 deacetylation assay with p53-substrate yielded only a minor inhibitory effect (Figure 2C), and in the more sensitive FdL assay, non-acetylated Tat-46–54 inhibited mini-Sirt1 only with low potency (IC_50_ 130.8 ± 88.1 µM; Supplementary Figure S1B). Thus, non-acetylated Tat-46–54 is not sufficient for potent Sirt1 inhibition.

We, then, tested the idea that initial Tat binding for potent inhibition requires Lys50 (de)acetylation. Using ac-Tat-46–54 as a substrate revealed efficient Sirt1-dependent deacetylation, even slightly more efficient than for its standard substrate peptide ac-p53-Lys382 (Figure 2D). We did not observe any inhibitory effects at later time points of the assays, as would have been expected if accumulating Sirt1-bound, deacetylated Tat-46–54 would inhibit (Supplementary Figure S2A). Consistently, adding ac-Tat-46–54 to an assay containing already 100 μM p53-peptide as substrate showed the expected increase of activity due to the increase in total substrate concentration, yet no indications of instant or delayed onset of inhibition (Figure 2E). These results indicate that Lys50 (de)acetylation is not relevant for potent inhibition.

**Scheme 1:**
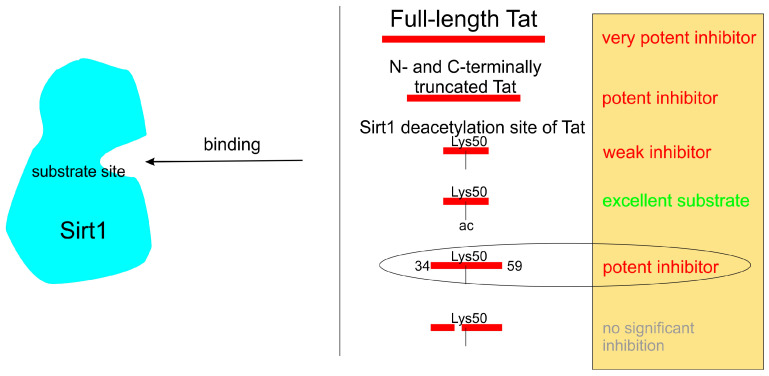
Schematic summary of the effects of different Tat fragments, with or without Lys50 acetylation, on the deacetylase activity of human Sirt1.

To analyze whether Tat residues outside the (de)acetylation region are relevant for inhibition, we next tested larger (non-acetylated) Tat fragments of the inhibitory region aa 20–63. We observed significant inhibition for Tat-31-60, weaker inhibition by Tat-49–60 and Tat-31–49, and no inhibitory effects for Tat-20–30 and Tat-35–44 (Figure 2F). The N-terminal region up to residue 30, and possibly even up to ~44, thus, is not relevant for Sirt1 inhibition. The regions 31–49 (C-terminal part thereof) and 49–60 both appear to contribute, indicating that either the region around 49 is critical or that both regions contain relevant residue areas, i.e., two residue patches are involved. We, thus, analyzed the inhibition of mini-Sirt1 through titrations with Tat-31–60, Tat-31–49, and Tat-49–60 (Figure 2G). While Tat-31–60 caused strong inhibition, neither Tat-31–49 nor Tat-49–60 alone was sufficient for significant inhibition of mini-Sirt1. Consequently, we focused on Tat peptides including regions both N- and C-terminal from the acetylation site Lys50. Tat-34–59, indeed, inhibited mini-Sirt1 with a potency similar to Tat-31–60 (Figure 2H). Shortening either N- or C-terminus in Tat-34–54 and Tat-37–59 reduced the potency (Figure 2H), yet still resulted in significant inhibition. We, therefore, used the Tat-37–59 fragment, comprising part of the Tat core and basic regions as well as the Sirt1 deacetylation site, as the Tat region essential for potent Sirt1 inhibition for further studies. The key results of these mapping studies are schematically summarized in Scheme 1.

### 2.3. Structural Basis of Tat-Dependent Sirtuin Inhibition

**Figure 3:**
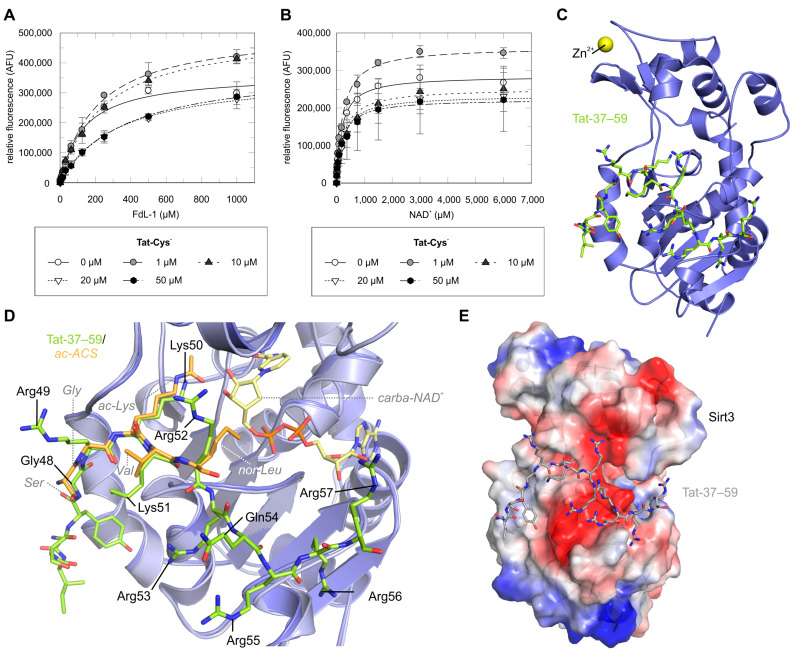
Mechanistic features of Tat-dependent sirtuin inhibition. (**A**) Michaelis–Menten kinetics for deacetylation of FdL-1 substrate by mini-Sirt1 in presence of increasing concentrations of Tat-Cys^−^ (error bars: s.d., *n* = 3). (**B**) Michaelis–Menten kinetics for the mini-Sirt1 cosubstrate NAD^+^ in presence of increasing concentrations of Tat-Cys^−^ (error bars: s.d., *n* = 3–6). (**C**) Sirt3 (blue) in complex with Tat-37–59 (lime). (**D**) Superposition of Sirt3/Tat-37–59 (blue/lime) with a Sirt3/ac-ACS/carba-NAD^+^ complex (light blue/orange; PDB ID 4FVT, RMSD = 0.5 Å for 242 C_α_ atoms). Residues of ac-ACS2 are labeled in italics. (**E**) Sirt3/Tat-37–59 with Sirt3 shown as surface colored according to electrostatic potential, from −62.5 k_b_T/e_c_ (red) to +62.5 k_b_T/e_c_ (blue).

To analyze the structural basis of Sirt1 inhibition by Tat, we tried to crystallize Sirt1 in complex with Tat-Cys^−^ and the inhibitory fragment 37–59. Since we failed to obtain a Sirt1 complex, we aimed to exploit our finding that Tat has comparable inhibitory effects on Sirt1–3 (Figure 1E). Analyzing the kinetic effects indeed showed that Tat inhibition is competitive with the acetylated substrate peptide and non-competitive with NAD^+^ for both Sirt1 and Sirt3 (Figure 3A,B and Supplementary Figure S2B,C), confirming a shared mechanism. We, thus, used Sirt3 as a model system for structural analysis of the sirtuin/Tat interaction and resolved the crystal structure of a Sirt3/Tat-37–59 complex at 1.95 Å resolution. The structure was refined to R_cryst_/R_free_ values of 22.7/27.6% and revealed clear electron density for part of the peptide ligand (Table 1 and Figure 3C and Supplementary Figure S2D). Consistent with the kinetic data, the inhibitory Tat fragment occupies the sirtuin substrate peptide channel but does not overlap with the NAD^+^ site (Figure 3C,D). Best defined in the inhibitory peptide is a small fragment, residues 48–52, which belongs to the Tat basic domain, while residues 44–47 and 53–57 are primarily defined via their peptide backbone (Supplementary Figure S2D). The complete basic motif assumes an extended conformation, terminated by Pro58. This conformation resembles the generic sirtuin–substrate interaction, which comprises a four-residue substrate stretch in β-strand conformation inserted between the β8-α9 loop and the β6-α8 loop of the sirtuin (Figure 3D) [12,28]. The sirtuin-bound Tat conformation also resembles the conformation of this Tat region in NMR structures of the apo forms of wt-Tat and Tat-Cys^−^ (Supplementary Figure S2E) [25], indicating that the appropriate conformation for sirtuin binding is intrinsically preferred and this Tat region, thus, “primed” for sirtuin binding. The Tat peptide assumes a binding mode deviating from substrates (see below), however, N- and C-terminally from this basic core, supported by charge complementarity (Figure 3E). While the N-terminus binds at a positively charged sirtuin surface patch, the C-terminal basic motif with its positive charges occupies a strongly negatively charged sirtuin pocket.

**Figure 4:**
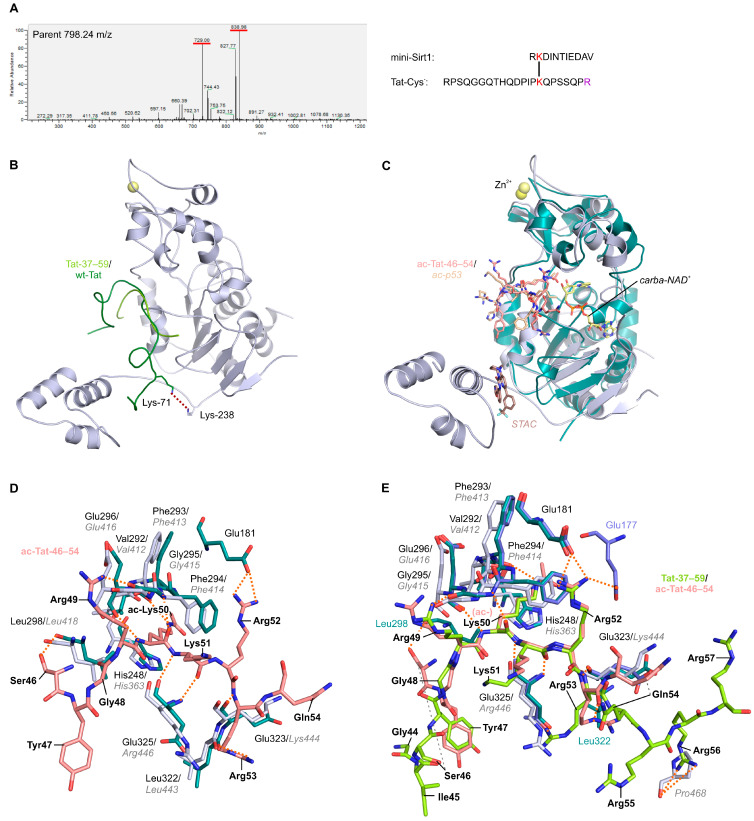
Structural basis of Tat deacetylation and Tat-dependent sirtuin inhibition. (**A**) MS^2^ spectrum of DSSO-interlinked peptides of mini-Sirt1 and Tat-Cys^−^ (parent 798.24 m/z). Selected ions for MS^3^ analysis are highlighted (red line). Tat Arg78 (purple) is deaminated. (**B**) Visualization of the crosslink between Tat-Lys71 and Sirt1-Lys238 (both Lys as sticks). An NMR structure of wt-Tat (dark green; PDB ID 1TBC) was superpositioned on Sirt3/Tat-37–59 and in turn overlaid with a mini-Sirt1/ac-p53/carba-NAD^+^/STAC complex (gray; PDB ID 4ZZJ). For clarity, only Sirt1 and Tat are shown. (**C**) Superposition of Sirt3/ac-Tat-46–54 (teal) with a mini-Sirt1/ac-p53/carba-NAD^+^/STAC complex (gray; PDB ID 4ZZJ, RMSD 1.2 Å for 208 C_α_ atoms). The ligands ac-Tat-46–54 (salmon), ac-p53 (light orange), carba-NAD^+^ (light yellow), and STAC (brown) are shown as sticks. (**D**) Interactions of ac-Tat-46–54 (salmon) with Sirt3 (teal), and Sirt1 overlaid in gray with their respective residues labeled in italics. Hydrogen bonds are shown as orange dotted lines. (**E**) Superposition of Sirt3/Tat-37–59 (blue) with Sirt3/ac-Tat-46–54 (teal, RMSD = 0.3 Å for 247 C_α_ atoms) and a mini-Sirt1/ac-p53/carba-NAD^+^/STAC complex (gray; PDB ID 4ZZJ, RMSD = 1.2 Å for 210 C_α_ atoms). Hydrogen bonds (orange dotted lines) and hydrophobic interactions of Tat-37–59 (lime) and ac-Tat-46–54 (salmon) with Sirt3 and the putatively involved residues in Sirt1 are labeled in italics.

To confirm that the binding mode observed in the Sirt3/Tat complex also applies to the Sirt1/Tat complex, we analyzed the Sirt1/Tat interaction by crosslinking. Incubation of mini-Sirt1/Tat-Cys^−^ with DSSO resulted in a strong band for a linked complex (Supplementary Figure S3A), and MS analysis after proteolysis revealed a crosslink between Tat-Lys71 and Sirt1-Lys238 (Figure 4A and Supplementary Figure S3B–F). Tat-Lys71 is not visible in our Sirt3/Tat complex, and therefore, we overlayed it with the NMR structure of apo wt-Tat, centered on Lys50, to visualize a larger Tat ligand. Superposition of this Tat complex with a Sirt1 substrate peptide complex reveals that Tat binding to Sirt1 in the same mode as observed in the Sirt3 complex would position Lys71 perfectly for the crosslink with Sirt1-Lys238 (Figure 4B). The crosslinking data, thus, support the substrate-like Tat-binding mode to Sirt1 and that it serves as the generic binding mode for sirtuin inhibition in solution.

To analyze why Tat inhibits sirtuins instead of serving as a regular substrate, we solved the crystal structure of a Sirt3/ac-Tat-46–54 complex at 1.65 Å resolution and refined it to R_cryst_/R_free_ values of 18.1/20.9% (Table 1 and Figure 4C and Supplementary Figure S3G). Residues 46–54 of the Tat substrate peptide are inserted in the substrate binding groove in the typical, β-sheet-like fashion observed in a previous Sirt1/ac-p53 peptide complex and other sirtuin/substrate complexes (Figure 4C). The acetylated Tat Lys50 interacts with Sirt3 Val292, Phe293/294, and the catalytic His248, and comparable interactions would be formed with Sirt1 (Figure 4D). In addition, Tat residues in the range of (−3) to (+3) relative to Lys50 interact, similarly, with Sirt3 (and likely Sirt1) in the complexes with acetylated and non-acetylated Tat, respectively (Figure 4D,E). Small differences in this range concern sidechain conformation, which does not affect directed interactions. Larger differences are seen for more remote residues, Ser46 at the N-terminus, and Gln54 at the C-terminus and indicate a different chain orientation from there on. In particular, the binding mode of the more C-terminal Tat residues comprising the basic region, which seems to be key in inhibitory Tat binding, could be influenced electrostatically by Lys50 acetylation, which removes a positive charge (Figure 4D,E). However, the molecular details that make sirtuin binding of deacetylated Tat inhibitory remain speculative and will require further studies of the complexes and interactions described here. In Sirt1, Tat residues C-terminal from the acetylation site (around Arg53) might, in addition, prevent positioning of the Sirt1 SBD on top of the active site (Figure 4E), possibly explaining the slightly more potent inhibition of this isoform (Figure 1D,E).

We, thus, conclude that Tat binds with a basic region core, residues 48–52, which comprise the Sirt1 substrate site Lys50, to the substrate binding site of Sirt1 and Sirt3 for competitive inhibition. Binding is supported by the extended conformation and the charge complementarity of this Tat region to the sirtuin substrate binding cleft. Inhibition does not require prior acetylation and Tat, thus, appears to act as a constitutive Sirt1 inhibitor, independent of its own acetylation status.

## 3. Discussion

We could show that inhibition of Sirt1 by HIV1 Tat neither requires a Tat-bound metal cofactor nor a Cys-mediated interaction with the Sirtuin Zn^2+^ cofactor. In fact, the N-terminal part of the Tat core, which complexes metal cofactors during interaction with p-TEFb [29], is not involved in Sirt1 binding. This is in contrast to a previous immunoprecipitation study that linked Tat’s Cys-rich and core regions to its Sirt1 interaction, using as a readout the hyperacetylation of the p65 subunit of NF-κB [9], which drives early phase HIV1 transcription [9,30]. The effects on p65 hyperacetylation in that study might be indirect and caused by the effects of the recruitment of p-TEFb to TAR and the dissociation of Tat from TAR rather than the direct Sirt1/Tat interaction. Our results show that the basic region of Tat plays a key role in the constitutive inhibition of Sirt1, independent of the acetylation status of Tat-Lys50. It binds substrate-like, in β-strand conformation, to the Sirt1 substrate cleft, supported by charge complementarity. We and others [8] observed that Tat can also inhibit Sirt2 and Sirt3 in vitro. These effects are unlikely to be physiologically relevant due to their subcellular localization mostly in the cytosol (Sirt2) and mitochondria (Sirt3), respectively [19], but they support the identified, generic binding mode, relying on the conserved substrate site geometry. The interaction based on the elongated conformation of the Tat basic region, similar to a Sirt1 substrate, has been observed for Tat in other interactions, e.g., with importin-α (PDB ID 5SVZ) [31] or PCAF (PDB ID 1JM4) [23]. The relevance of this Tat region for Sirt1 inhibition is illustrated by the higher potency of Tat peptides that include residues 55–59. Our structural studies show that binding of this region to sirtuins leads to subtle differences compared to a substrate, likely driven by electrostatics, leading to the different functional outcomes of inhibition, respectively, deacetylation. The role of electrostatics is supported by our finding that Sirt5 and Sirt6 are insensitive to Tat inhibition. These isoforms are unique in their substrate binding clefts comprising mostly hydrophobic and positively charged patches, reflected by their preference for hydrophobic and negatively charged substrate residues downstream from the deacylation site [13]. Sirt1–3, instead, prefer positively charged residues downstream from the acetylation site and are, therefore, amenable to binding the Tat basic region to this cleft. Interestingly, the binding mode of the Tat residues C-terminal to Gln54 could be influenced electrostatically by Lys50 acetylation, which eliminates a positive charge. Our finding that Sirt1 inhibition is independent of Lys50 acetylation indicates, however, that the role of this (de)acetylation is to influence other Tat functions than Sirt1 inhibition, most likely the interaction with the p-TEFb/TAR complex. Our data, indeed, indicate that Tat binds in a slightly different conformation compared to a substrate and that Tat serving as a substrate has to dissociate before it can bind in the inhibitory mode.

Both Tat interactions with Sirt1, as a substrate and as an inhibitor, are relevant during HIV1 transcription. Sirt1 inhibition is necessary to drive NF-κB-mediated HIV1 transcription during the early phase of infection, while Sirt1-dependent Tat deacetylation promotes its release from PCAF, freeing up Tat for binding to TAR, and thereby, initiating a new cycle of viral transcription [9,30]. We find that Tat inhibits Sirt1 independent from its own acetylation, although technical difficulties (recombinant production of sufficient amounts of specifically acetylated full-length Tat) precluded confirmation of this finding from peptide studies with full-length Tat. We further find that Tat binds to the Sirt1 substrate site not only for its own deacetylation but also for competitive Sirt1 inhibition, showing that Tat exerts its two functional roles via the same binding site. Thus, it remains enigmatic as to why acetylated Tat can serve as a substrate without substantial inhibition from the formed product, deacetylated Tat, while deacetylated Tat newly encountering Sirt1 causes inhibition. Further studies will be required to fully understand the mechanistic details of this functional switch and its regulation.

## 4. Conclusions

The HIV1 protein Tat is part of complex regulation machinery that adapts cellular metabolism to the needs of the virus. Tat function and regulation include two types of interaction with Sirt1. First, as a Sirt1 inhibitor to enable early viral genome transcription and possibly a weakening of the host immune response. Second, as a Sirt1 substrate for deacetylation at Lys-50, which modulates Tat’s interactions with other regulatory complexes, and thereby, supports the re-initiation of viral genome transcription. In our study, we could map the part of Tat that mediates Sirt1 inhibition to its core and basic region, which included the Sirt1 deacetylation site Lys50, and we observed that Tat inhibits Sirt1 via competition with the deacetylation substrate (Scheme 1). Moreover, we could show that Tat, indeed, binds to the sirtuin catalytic core in a substrate-like, extended fashion, exploiting its intrinsically extended basic region for binding to the sirtuin substrate-binding cleft. Although it is not acetylated, inhibitory Tat Lys50 is positioned in the sirtuin substrate lysine pocket. The Sirt1/Tat interaction is modulated, however, by a charge complementarity that leads to a partial isoform selectivity and a binding mode slightly deviating from a regular substrate complex, rationalizing the substrate-competitive inhibitory effect. Importantly, we find that binding and inhibition appear not to be facilitated by prior acetylation and Sirt1-dependent deacetylation of Tat, showing that acetylated Tat serves only as a substrate, and not as a mechanism-based inhibitor that gets activated by turnover and remains bound as an inhibitor. Instead, deacetylated Tat has to accumulate to rebind, in the inhibitory mode, to the Sirt1 substrate site. Therefore, fluctuations of Tat (de)acetylation would help to fine-tune viral transcription processes in an autoregulatory loop with Sirt1. Thus, our study improved our understanding of physiological Sirt1 regulation and the role of the Sirt1/Tat interaction during HIV1 infection, and it provided a structural basis for this regulation mechanism that will enable further studies to fully resolve its mechanistic details.

## 5. Methods

### 5.1. Chemicals

If not stated otherwise, chemicals were purchased from Sigma-Aldrich (Saint Louis, MO, USA).

### 5.2. Expression and Purification of Proteins

A human mini-Sirt1 construct (aa 183-505-(GGGS)_2_-641-665), as described in [27], was generated in a modified pET-19b with a TEV cleavage site. This construct and human full-length Sirt1 (fl-Sirt1) in pET-15b, Sirt1-(183-664) and Sirt1-(214-664) in pET-15b, mini-Sirt1-∆SBD (aa 214-505-(GGGS)_2_-641-665) and mini-Sirt1-∆ESA (aa 183–505) in a modified pET-19b with TEV cleavage site, Sirt2-(43-356) in a modified pET-19b with a His_6_-Sumo tag, Sirt3-(93-399) in a modified pET-32a with Trx-His_6_ tag and TEV cleavage site, hSirt3-(118-399) in pVFT3S (Sungkyunkwan University, Seoul, Republic of Korea) with His_6_-Trx tag and TEV cleavage site, Sirt5-(34-302) in pET-151/D-TOPO, and Sirt6-(1-355) in pQE80L.1 were purified as described [32,33,34]. Shortly, proteins were expressed at 20 °C (mini-Sirt1 constructs at 16 °C) in *E. coli* CodonPlus(DE3), cell lysates were cleared by centrifugation, and proteins were purified via affinity chromatography using a nickel NTA column. After digestion with thrombin or TEV protease, proteins were re-run over the nickel NTA column, pooled, and concentrated. The samples were, then, subjected either directly to size exclusion chromatography in 50 mM Tris/HCl, pH 8.0, 150 mM NaCl, 1 mM TCEP, or to an ion exchange chromatography followed by size exclusion chromatography. Purified proteins (yield 1—4 mg/L expression culture) were concentrated to 12–18 mg/mL and shock frozen for storage at −80 °C.

Tat-Cys^−^-(1–86) were expressed in *E. coli* BL21 (DE3) using a pET11a vector (Novagen) harboring the HIV1 Zaire 2 tat gene with amino acid exchanges C22S, C25A, C27A, C30S, C31A, C34S, and C37A [25]. Cells grown for 4 h at 37 °C were harvested and resuspended in 50 mM Tris–HCl pH 8.0, 1 M MgCl_2_, 200 mM NaCl, 2 mM phenylmethylsulfonylfluoride (PMSF), a protease inhibitor mix (Roche, Basel, Switzerland), DNase I, and benzamidine [35]. After 30 min on ice, cells were lysed with a Microfluidizer (MFTI Corporation, Manila, Philippines), the pH slowly decreased to pH 3–4 with 1 M HCl, stirred for 45 min on ice, and centrifuged for 30 min at 4 °C and 16,503× *g*. Then, the pH was increased to pH 8 and the protein precipitated by slowly adding 0.436 g (NH_4_)_2_SO_4_ per mL, and stirring for 15 min at room temperature, followed by 2 h stirring at 8 °C, and centrifugation for 30 min at 4 °C and 16,503× *g*. The pellet was resuspended in 10 mM potassium phosphate pH 6.4, 200 mM NaCl and protease inhibitor mix, and dialyzed overnight against 20 mM potassium phosphate, pH 6.4, 200 mM NaCl, and 2 mM PMSF. The solution was centrifuged for 20 min at 4 °C and 19,000× *g* and the supernatant was applied to a HeparinHP column (GE Healthcare, Chicago, IL, USA). The protein was eluted using a NaCl gradient from 200 mM to 2 M NaCl. Tat-Cys^−^ fractions were pooled, dialyzed twice against 10 mM potassium phosphate pH 6.4, 150 mM NaCl, and concentrated to 7 mg/mL Tat-Cys- with a 5 kDa VivaSpin20 concentrator (Sartorius; yield ~4–6 mg protein/l expression culture). Aliquots were shock frozen in liquid nitrogen and stored at −80 °C.

### 5.3. NMR Spectroscopy

NMR titration experiments were performed by stepwise addition of unlabeled mini-Sirt1 (stock concentration 260 µM) to 100 µM ^15^N-labeled Tat-Cys^−^ in 150 mM sodium chloride, and 10 mM potassium phosphate (pH 6.4). Spectra for 2D-(^1^H, ^15^N)-HSQC NMR were recorded at 298 K on a Bruker 700 MHz spectrometer equipped with a cryogenically-cooled probe.

### 5.4. Peptide- and FdL-Based Sirtuin Activity Assays

For sirtuin deacetylation activity measurements, we used two well-characterized assays, a coupled enzymatic assay, and the so-called FdL assay, respectively [17].

Continuous coupled enzymatic sirtuin activity assays were conducted as described [36]. Briefly, assays in 10 mM potassium phosphate pH 7.8 contained 1 µM sirtuin, 2 µM nicotinamidase, 2 U/mL GDH, 3.5 mM α-ketoglutarate, 0.5 mM NADPH, 0.5 mM NAD^+^, and peptide substrate at 100 µM, or at the respective peptide substrate’s K_m_ if indicated. K_m_ values for peptide substrates (mini-Sirt1: 200 µM ac-p53, hSirt2: 95 µM α-tubulin, 150 µM ac-p53, hSirt3: 250 µM ac-ACS2, hSirt5: 23 µM succ-CPS1, hSirt6: 5 µM myr-TNFα) were determined in the presence of 3 mM NAD^+^. For assays with Tat peptides, 5% (*v*/*v*) DMSO was added to all reactions to enhance peptide solubility. Reactions were monitored for 1 h at room temperature by absorption spectroscopy at 340 nm in a UV/vis plate reader (BioTek). The slope for the linear range (typically 5–25 min) was evaluated after background correction with measurements without NAD^+^. Additional control reactions were run by either omitting enzyme or omitting peptide substrate to ensure that the signal measured reflects the NAD+-dependent deacetylation activity of the sirtuin.

FdL assays were conducted at 37 °C in 10 mM potassium phosphate, pH 7.8 with 1 µM sirtuin, 500 µM NAD^+^, and 100 µM FdL-1 substrate, or at the respective FdL-1 K_m_ (fl-Sirt1: 230 µM, mini-Sirt1: 144 µM, Sirt2: 300 µM, Sirt3: 135 µM) if indicated. K_m_ values for FdL-1 were determined in the presence of 3 mM NAD^+^ and K_m_ values for NAD^+^ in presence of 0.5 mM FdL-1 peptide. For assays with Tat peptides, 15% (*v*/*v*) DMSO (or 20% (*v*/*v*) DMSO for Tat-34-54, Tat-34-59, and Tat-37-54) was added to enhance peptide solubility. Reactions were incubated for 20 min, followed by a 1:1 addition of developer (2 mM NAM and 10 mg/mL trypsin in assay buffer). After incubation for 45 min at room temperature, fluorescence was measured with a FluoDia T70 (Photon Technology International Inc., Birmingham, NJ, USA) with an excitation wavelength of 365 nm and emission wavelength of 465 nm. Background corrections were performed with measurements without sirtuin, and controls were run without NAD+ or FdL peptide, respectively. To exclude the Tat peptides from having any effects on the FdL-1 substrate, additional control reactions were run at 37 °C, stopped by the addition of developer, and inhibitory peptides were, subsequently, added before the signal was recorded.

### 5.5. Crosslinking and Mass Spectrometric Analysis

Prior to crosslinking, mini-Sirt1, and Tat-Cys^−^ were dialyzed separately at 4 °C in Xpress Micro Dialyzer tubes (Serva, MD300, cutoff 6–8 kDa) against 25 mM HEPES, pH 7.5, 150 mM NaCl, 5% (*v*/*v*) glycerol. Then, 50 µM of each protein was crosslinked at 20 °C with 2.82 mM disuccinimidyl sulfoxide (DSSO) in 5% (*v*/*v*) DMSO. Before the addition of the crosslinker, 10 µL was removed from the reaction (t = 0 min) and stopped by adding 1 µL 1 M Tris/HCl, pH 8.0. Likewise, at several timepoints after the addition of DSSO, 10 µL of the reaction was sampled. Reaction samples were supplemented with SDS sample buffer and separated on a 15% SDS-PAGE. Controls contained only one of the two proteins and were treated similarly to exclude self-crosslinking.

Crosslinked protein bands were cut out from SDS-PAGE gels and digested with 200 ng trypsin in 25 mM NH_4_HCO_3_, pH 8.0, at 37 °C overnight. The digested proteins were dried in a vacuum centrifuge, resuspended in 0.1% (*v*/*v*) FA, and injected onto a desalting column, followed by automated peptide separation with a C18 column (buffer A: 0.1% (*v*/*v*) FA, 5% (*v*/*v*) acetonitrile; buffer B: 0.1% (*v*/*v*) FA, 95% (*v*/*v*) acetonitrile). MS/MS spectra were recorded with an LTQ mass spectrometer (Thermo Fischer Scientific, Waltham, MA, USA) and CID-cleaved inter-protein crosslinks were analyzed with Byonic^TM^ (Protein Metrics Inc., San Carlos, CA, USA).

### 5.6. Crystallization and Structure Determination of Sirtuin Complexes

For crystallization of the Sirt3 and Tat peptide complexes, 10 mg/mL human Sirt3-(118-399) in 20 mM Tris/HCl, pH 8.0, 150 mM NaCl, 5% (*v*/*v*) glycerol, 1 mM TCEP were incubated with 2 mM ac-Tat-46-54 or Tat-37-59 (the latter in 10% (*v*/*v*) DMSO) for 60 min at 20 °C. Both complexes were crystallized using the sitting-drop vapor-diffusion method at 20 °C with 100 mM MES, pH 6.0, 30% (*w*/*v*) PEG 200, 5% (*w*/*v*) PEG 3000 as reservoir solution for Sirt3/ac-Tat-46-54 and 100 mM CHES, pH 9.0, 20% (*w*/*v*) PEG 8000 for Sirt3/Tat-37-59. Crystals were cryoprotected with the respective reservoir solution supplemented with 25% (*v*/*v*) glycerol and flash-frozen in liquid nitrogen.

Diffraction data were collected at BESSY II, beamline MX14.1 operated by Helmholtz Zentrum Berlin (Berlin, Germany) at 0.9184 Å wavelength. Indexing, scaling, and merging of the Sirt3/ac-Tat-46-54 complex data were performed with XDSAPP [37]. The Sirt3/Tat-37–59 complex data were indexed and integrated with DIALS [38] and further processed with CCP4 programs Rebatch, Pointless, Aimless, and cTruncate, with 5% of the reflections assigned to R_free_ with Uniqueify [39]. The complex structures were phased by molecular replacement with PHASER [40] and using a Sirt3/ac-ACS/carba-NAD (PDB ID 4FVT) and a Sirt3/ADPr complex (PDB ID 4BVB), respectively, as a search model. Structures were refined with individual B-factors in Phenix [41] and rebuilt and validated using Coot [42]. Structure figures were generated with PyMOL (Schrödinger LLC, New York City, NY, USA).

## Figures and Tables

**Table 1:** Diffraction data and refinement statistics.

	Sirt3/Tat-37–59	Sirt3/ac-Tat-46–54
Resolution ^(a)^	54.24–1.95 (2.02–1.95)	39.36–1.65 (1.71–1.65)
Space group	P2_1_	C222_1_
Unit cell constants	a = 54.5 Å, b = 78.1 Å, c = 76.6 Å α = 90.0°, β = 96.1°, γ = 90.0°	a = 78.7 Å, b = 127.8 Å, c = 77.6 Å α = β = γ = 90.0°
Unique reflections ^(a)^	46,174 (4615)	47,332 (4698)
Multiplicity ^(a)^	5.5 (5.5)	11.2 (11.2)
Completeness ^(a)^	98.8% (98.9%)	99.9% (99.9%)
I/σ(I) ^(a)^	12.0 (0.6)	13.9 (0.9)
R_meas_ ^(a)^	29.9% (160.4%)	9.5% (22.6%)
CC_1/2_ ^(a)^	0.95 (0.45)	1.00 (0.47)
Protein atoms	4563	2244
Ligand atoms	2	1
Solvent atoms	472	287
R_cryst_/R_free_	22.7%/27.6%	18.1%/20.9%
Average B-factors		
Protein	38.6	35.6
Ligands	25.9	26.9
Solvent	41.3	47.1
RMSD bond lengths	0.002 Å	0.017 Å
RMSD bond angles	0.520°	1.380°
Ramachandran		
Favored	97.2%	98.9%
Allowed	2.8%	1.1%
Outliers	0.0%	0.0%

^(a)^ Values in parentheses refer to the outermost shell.

## Data Availability

Structure factors and refined structures have been deposited with the Protein Data Bank (http://www.rcsb.org/pdb) under accession codes 8CCW (Sirt3/ac-Tat-46–54) and 8CCZ (Sirt3/Tat-37–59).

## Supplementary Materials

The following supporting information can be downloaded at: https://www.mdpi.com/article/10.3390/life13040949/s1: Supplementary Figure S1–S3.

## Abbreviations

AIDS	acquired immunodeficiency syndrome
ARM	arginine-rich motif
AROS	active regulator of Sirt1
Dbc1	deleted in breast cancer 1
DSSO	disuccinimidyl sulfoxide
ESA	essential for Sirt1 activity
FdL	Fluor-de-Lys
fl-Sirt1	full-length sirtuin 1
GDH	glutamate dehydrogenase
HIC1	hypermethylated in cancer 1
HIV1	human immunodeficiency virus 1
NAD^+^	nicotinamide adenine dinucleotide
NAM	nicotinamide
PCAF	p300/CBP-associated factor
PMSF	phenylmethylsulfonyl fluoride
p-TEFb	positive-acting transcription elongation factor b
SBD	STAC-binding domain
STAC	sirtuin-activating compound
TAR	transactivation response
Tat	transactivator of transcription
