# Supplementary material for: Molecular Mechanism of Sirtuin 1 Inhibition by Human Immunodeficiency Virus 1 Tat Protein
Source: Life (Basel). 2023 Apr 4;13(4):949. doi: 10.3390/life13040949 (PMC10144703; doi:10.3390/life13040949)
Supplement: Supplementary file 1 [file life-13-00949-s001.zip › life-2225018-supplementary.pdf]

Supplementary Information

Molecular mechanism of Sirtuin 1 inhibition by human immunodeficiency virus 1 Tat protein

Ramona S. Adolph[a], Eileen Beck[a], Kristian Schweimer[b], Andrea Di Fonzo[a], Michael Weyand[a], Paul Rösch[b], Birgitta M. Wöhrl[b], Clemens Steegborn[a]*

[a] Department of Biochemistry, University of Bayreuth, 95440 Bayreuth, Germany

[b] Department of Biopolymers, University of Bayreuth, 95440 Bayreuth, Germany

*** Correspondence:** Clemens Steegborn, University of Bayreuth, Department of Biochemistry, Universitätsstr. 30, 95447 Bayreuth, Germany; phone: (+49)(921)557831; fax: (+49)(921)557832; email: Clemens.Steegborn@uni-bayreuth.de

Supplementary Figure 1

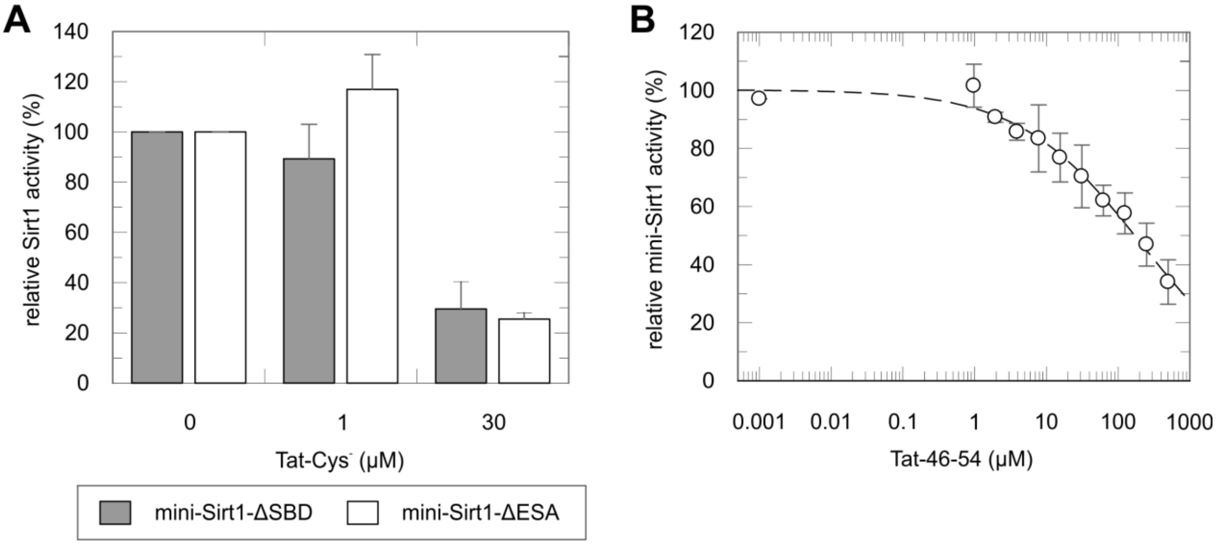

Supplementary Figure S1: Mapping of the interaction sites on Sirt1 and Tat. (A) Dose-dependent inhibition of mini-Sirt1 deletion constructs by Tat-Cys$^-$ in presence of 100 µM ac-p53. (error bars: s.d., n=3) **(B)** Titration of mini-Sirt1 with non-acetylated Tat-46-54 in an FdL assay at substrate K_m. (error bars: s.d., n=3)

Supplementary Figure 2

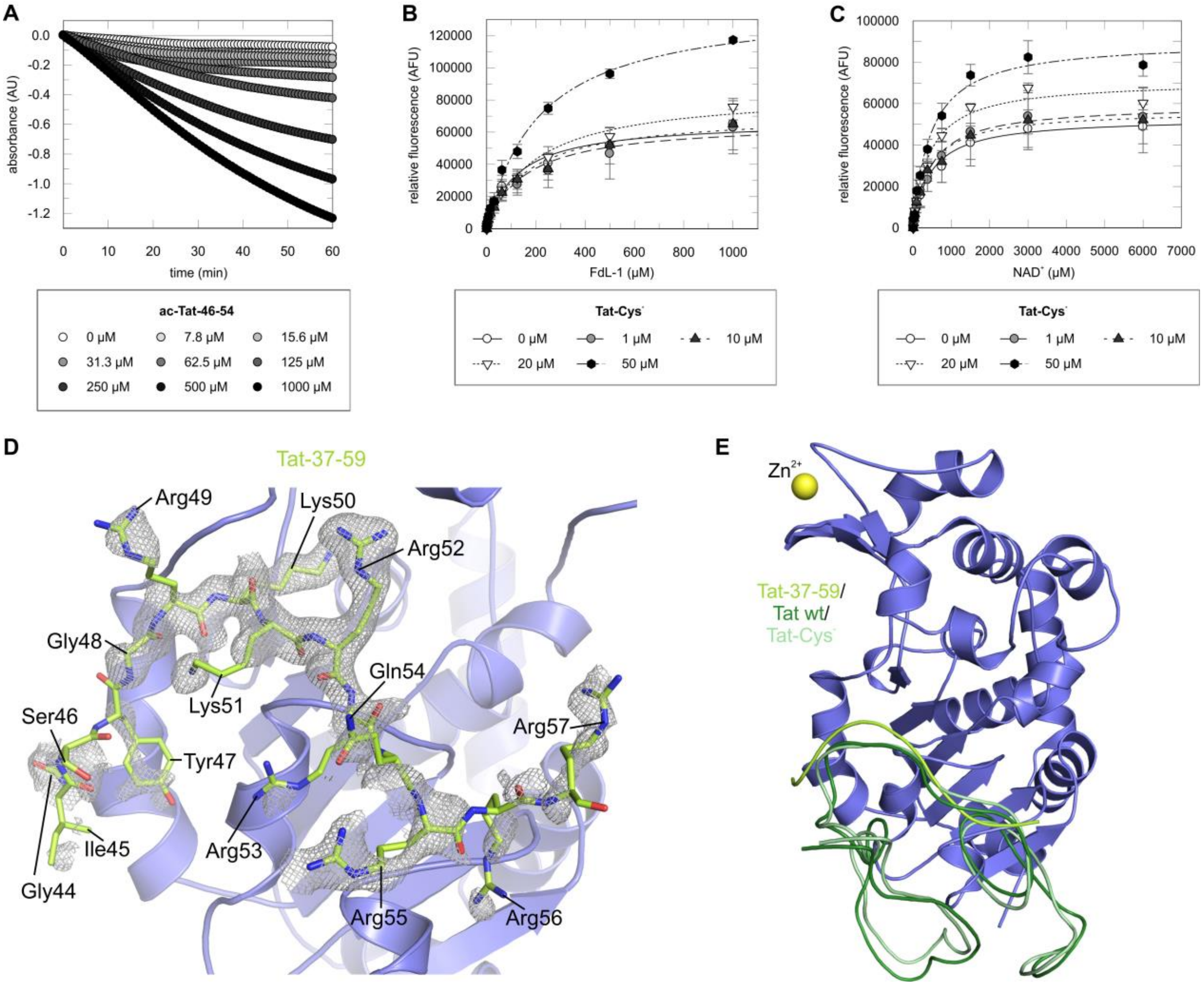

Supplementary Figure S2: Kinetic mechanism and structural basis of Tat deacetylation and Tat-mediated Sirtuin inhibition. (A) Time-course for deacetylation of ac-Tat-46-54 by mini-Sirt1. (n=3) **(B)** Deacetylation of FdL-1 substrate by Sirt3-(93-399) in presence of increasing concentrations of Tat-Cys$^-$. (error bars: s.d., n=3-6). **(C)** Titrations of Sirt3-(93-399) with the cosubstrate NAD^+ in presence of increasing concentrations of Tat-Cys$^-$. (error

bars: s.d., n=3-6). **(D)** Crystal structure of Sirt3 (blue) in complex with Tat-37-59 (lime). The $2mF_o$-DF_c composite omit electron density map is contoured at 1σ. **(E)** Superposition of the crystal structure of Sirt3/Tat-37-59 with NMR structures of wildtype Tat (dark green; PDB: 1TBC, RMSD = 3.996 Å for 14 C_α atoms) and Tat-Cys$^-$ (light green; PDB: 1TAC, RMSD = 4.029 Å for 14 C_α atoms).

Supplementary Figure 3

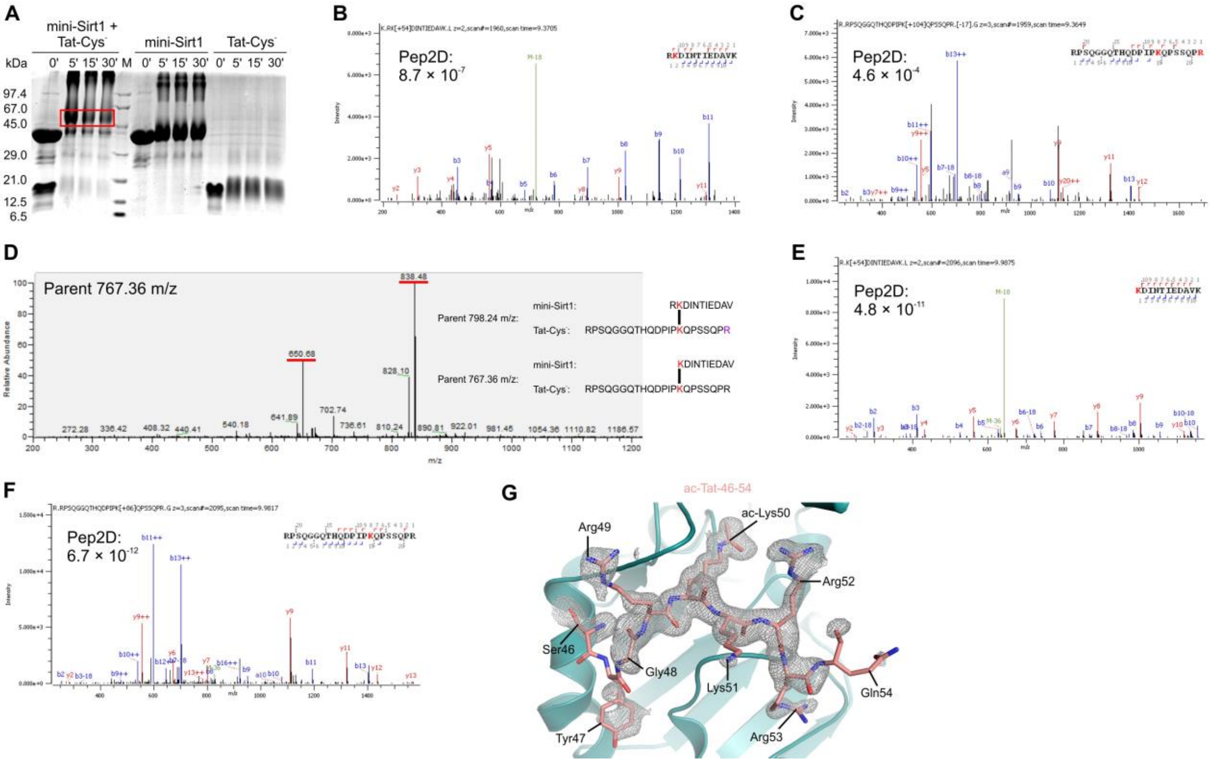

Supplementary Figure S3: Crosslinking of Sirt1 and Tat. (A) SDS-PAGE of DSSO-crosslinked mini-Sirt1 and Tat-Cys^{-}. The interprotein crosslink is highlighted with a red box. **(B)** MS^3 spectrum of 729.00 m/z including the mini-Sirt1 peptide sequence and Pep2D value (parent 798.24 m/z). **(C)** MS^3 spectrum of 838.98 m/z including the Tat-Cys^{-} peptide sequence and Pep2D value (parent 798.24 m/z). **(D)** MS^2 spectrum of DSSO-interlinked peptides of mini-Sirt1 and Tat-Cys^{-} (parent 767.36 m/z). Selected ions for MS^3 analysis are highlighted (red line). **(E)** MS^3 spectrum of 650.68 m/z including the mini-Sirt1 peptide sequence and Pep2D value. **(F)** MS^3 spectrum of 838.48 m/z including the Tat-Cys^{-} peptide sequence and Pep2D value. **(G)** Crystal structure of Sirt3 (teal) in complex with ac-Tat-46-54 (salmon). The $2mF_o$-DF_c composite omit electron density map is contoured at 1σ.
